# Cdk5-mediated CRMP2 phosphorylation is necessary and sufficient for peripheral neuropathic pain

**DOI:** 10.1016/j.ynpai.2018.07.003

**Published:** 2018-07-26

**Authors:** Aubin Moutal, Shizhen Luo, Tally M. Largent-Milnes, Todd W. Vanderah, Rajesh Khanna

**Affiliations:** a Department of Pharmacology, College of Medicine, University of Arizona, Tucson, AZ 85724, USA; b Department of Anesthesiology, College of Medicine, University of Arizona, Tucson, AZ 85724, USA; c Neuroscience Graduate Interdisciplinary Program, College of Medicine, University of Arizona, Tucson, AZ 85724, USA; d The Center for Innovation in Brain Sciences, University of Arizona, Tucson, AZ 85724, USA

**Keywords:** Spared nerve injury, Neuropathic pain, CRMP2, Cyclin-dependent kinase 5, Phosphorylation

## Abstract

Neuropathic pain results from nerve injuries that cause ectopic firing and increased nociceptive signal transmission due to activation of key membrane receptors and channels. The dysregulation of trafficking of voltage-gated ion channels is an emerging mechanism in the etiology of neuropathic pain. We identify increased phosphorylation of collapsin response mediator protein 2 (CRMP2), a protein reported to regulate presynaptic voltage-gated calcium and sodium channels. A spared nerve injury (SNI) increased expression of a cyclin dependent kinase 5 (Cdk5)-phosphorylated form of CRMP2 in the dorsal horn of the spinal cord and the dorsal root ganglia (DRG) in the ipsilateral (injured) versus the contralateral (non-injured) sites. Biochemical fractionation of spinal cord from SNI rats revealed the increase in Cdk5-mediated CRMP2 phosphorylation to be enriched to pre-synaptic sites. CRMP2 has emerged as a central node in assembling nociceptive signaling complexes. Knockdown of CRMP2 using a small interfering RNA (siRNA) reversed SNI-induced mechanical allodynia implicating CRMP2 expression as necessary for neuropathic pain. Intrathecal expression of a CRMP2 resistant to phosphorylation by Cdk5 normalized SNI-induced mechanical allodynia, whereas mimicking constitutive phosphorylation of CRMP2 resulted in induction of mechanical allodynia in naïve rats. Collectively, these results demonstrate that Cdk5-mediated CRMP2 phosphorylation is both necessary and sufficient for peripheral neuropathic pain.

## Highlights

• CRMP2 phosphorylation levels are dysregulated in the SNI model of experimental neuropathy.
• CRMP2 phosphorylation by Cdk5 is increased at the pre-synaptic sites of the dorsal horn of the spinal cord.
• CRMP2 expression is necessary for neuropathic pain.
• Genetic targeting of CRMP2 phosphorylation by Cdk5 reverses neuropathic pain.
• CRMP2 phosphorylation by Cdk5 is sufficient to elicit allodynia.

## 1 Introduction

In most neuropathic pain patients, chronic pain develops after a partial nerve injury (Baron, 2000). Injury to a peripheral nerve results in a pathological state of sensory neurons leading to ectopic firing and increased nociceptive signal transmission (Wall and Gutnick, 1974). Activation of key membrane receptors and channels contributes to the neuronal plasticity and signal transduction changes in nociceptive neurons that initiate and maintain pathological pain states. The trafficking of voltage-gated ion channels (VGICs) appears to be a central mechanism in the etiology of neuropathic pain. VGICs regulate the generation of action potentials and the release of neurotransmitters (Tibbs et al., 2016). In neuropathic pain, the expression of proteins regulating the trafficking of voltage gated sodium channels (VGSC) is dysregulated (Berta et al., 2008, Laedermann et al., 2013). In particular, upregulation of the VGSC β-subunits (Berta et al., 2008) and downregulation of Nedd4-2 (an E3 ubiquitin ligase) (Laedermann et al., 2013) following a spared nerve injury (SNI) (Decosterd and Woolf, 2000), converge to functionally upregulate the voltage-gated sodium channel NaV1.7. Increased trafficking of voltage gated calcium channels (VGCCs) has also been reported in neuropathic pain (Dolphin, 2016). The pre-synaptic localization of the N-type (CaV2.2) VGCC is increased following a neuropathic pain injury (Cizkova et al., 2002). This has been related to the upregulation of the expression of the α2δ-1 subunit of VGCCs in neuropathic pain (Bauer et al., 2009). Furthermore, alternative splicing events render CaV2.2 insensitive to morphine induced G-coupled protein receptor downregulation (Jiang et al., 2013). These examples identify dysregulation of trafficking of NaV1.7 and CaV2.2 as a major pathological event contributing to neuropathic pain. In this context, we recently identified a novel protein – collapsin response mediator protein 2 (CRMP2) – having the ability to regulate both channels in chronic pain (Brittain et al., 2011, Chi et al., 2009, Dustrude et al., 2016, Dustrude et al., 2013, Francois-Moutal et al., 2015, Piekarz et al., 2012, Ripsch et al., 2012, Wang et al., 2010, Wilson et al., 2011).

CRMP2 was first identified as an axonal growth and guidance protein (Goshima et al., 1995, Inagaki et al., 2001). CRMP2’s cellular functions including neurite outgrowth, endocytosis, and ion channel trafficking (Brittain et al., 2011, Brustovetsky et al., 2014, Dustrude et al., 2013, Moutal et al., 2014) are heavily dependent on its multiple phosphorylation by cyclin-dependent kinase 5 (Cdk5) (Cole et al., 2006), glycogen synthase kinase 3β (GSK3β) (Yoshimura et al., 2005), Rho-associated protein kinase (ROCK) (Arimura et al., 2000), Yes (Varrin-Doyer et al., 2009), or the Src-family kinase Fyn (Uchida et al., 2009). Several studies identified CRMP2 phosphorylation by Cdk5 as a central event regulating subsequent phosphorylation by GSK3β (Cole et al., 2006) and SUMOylation (addition of small ubiquitin like modifier; SUMO) (Dustrude et al., 2016). In a genomically edited model of hyperalgesia, we identified CRMP2 phosphorylation by Cdk5 as a principal controller of trafficking of both CaV2.2 and NaV1.7 channels (Moutal et al., 2017). These findings were supported by other studies that underscored a key role of CRMP2 phosphorylation for CaV2.2 (Brittain et al., 2012, Moutal et al., 2016, Moutal et al., 2016;1(1)., Moutal et al., 2016) and NaV1.7 function (Dustrude et al., 2016). We further identified CRMP2 SUMOylation, an event primed by Cdk5 phosphorylation of CRMP2, to be upregulated in neuropathic pain (Moutal et al., 2017) and to contribute to nociceptive signal transmission by positive regulation of NaV1.7 trafficking (Moutal et al., 2017, Dustrude et al., 2013, Dustrude et al., 2016, Dustrude et al., 2017).

Here, we hypothesized that CRMP2 phosphorylation levels were dysregulated in neuropathic pain, thus leading to aberrant nociceptive signal transmission. We characterized the pattern of CRMP2 phosphorylation in the primary afferents after a peripheral nerve injury. Increased CRMP2 phosphorylation by Cdk5 was consistently found in all tissues tested. Expressing a CRMP2 mutated on the Cdk5 phosphorylation site reversed mechanical allodynia in a spared nerve injury (SNI) model of peripheral neuropathy. These results identify CRMP2 phosphorylation by Cdk5 as a major pathological event underlying neuropathic pain.

## 2 Methods

### 2.1 Animals

Pathogen-free, adult male Sprague–Dawley rats (250 g; Envigo) were housed in temperature (23 ± 3 °C) and light (12-h light/12-h dark cycle; lights on 07:00–19:00) controlled rooms with standard rodent chow and water available ad libitum. The Institutional Animal Care and Use Committee of the College of Medicine at the University of Arizona approved all experiments. All procedures were conducted in accordance with the Guide for Care and Use of Laboratory Animals published by the National Institutes of Health and the ethical guidelines of the International Association for the Study of Pain. Animals were randomly assigned to treatment or control groups for the behavioral experiments. Animals were initially housed three per cage but individually housed after the intrathecal cannulation on a 12 h light-dark cycle with food and water ad libitum. All behavioral experiments were performed by experimenters who were blinded to the experimental groups and treatments.

### 2.2 Spared nerve injury (SNI)

Under isoflurane anesthesia (5% induction, 2.0% maintenance in 2 L/min air), skin on the lateral surface of the left hind thigh was incised. The biceps femoris muscle was bluntly dissected to expose the three terminal branches of the sciatic nerve (Decosterd and Woolf, 2000). Briefly, the common peroneal and tibial branches were tightly ligated with 4-0 silk and axotomized 2.0 mm distal to the ligation. Sham animals underwent the same operation; however the exposed nerves were not ligated. Closure of the incision was made in two layers. The muscle was sutured once with 5-0 absorbable suture; skin was auto-clipped. Animals were allowed to recover for 7 days before any testing.

### 2.3 Immunoblot preparation and analysis

Tissue lysates prepared from adult Sprague-Dawley rats (day 12 after SNI) were generated by homogenization and sonication in RIPA buffer (50 mM Tris-HCl, pH 7.4, 50 mM NaCl, 2 mM MgCl_2_, 1% [vol/vol] NP40, 0.5% [mass/vol] sodium deoxycholate, 0.1% [mass/vol] SDS) as described previously (Francois-Moutal et al., 2015). Protease inhibitors (Cat# B14002; Bimake, Houston, TX), phosphatase inhibitors (Cat# B15002, Bimake), and benzonase (Cat#71206, Millipore, Billerica, MA). Protein concentrations were determined using the BCA protein assay (Cat# PI23225, Thermo Fisher Scientific, Waltham, MA). Indicated samples were loaded on 4–20% Novex® gels (Cat# EC60285BOX, Thermo Fisher Scientific, Waltham, MA). Proteins were transferred for 1 h at 120 V using TGS (25 mM Tris pH = 8.5, 192 mM glycine, 0.1% (mass/vol) SDS), 20% (vol/vol) methanol as transfer buffer to polyvinylidene difluoride (PVDF) membranes 0.45 μm (Cat# IPVH00010, Millipore, Billerica, MA), pre-activated in pure methanol. After transfer, the membranes were blocked at room temperature for 1 h with TBST (50 mM Tris-HCl, pH 7.4, 150 mM NaCl, 0.1% Tween 20), 5% (mass/vol) non-fat dry milk, then incubated separately in indicated primary antibodies: see Table 1) in TBST, 5% (mass/vol) BSA, overnight at 4 °C. Following incubation in horseradish peroxidase-conjugated secondary antibodies from Jackson immunoresearch, blots were revealed by enhanced luminescence (WBKLS0500, Millipore, Billerica, MA) before exposure to photographic film. Films were scanned, digitized, and quantified using Un-Scan-It gel version 6.1 scanning software by Silk Scientific Inc. For all experiments, CRMP2 phosphorylation levels were always normalized to total CRMP2 levels in the same sample.

**Table 1:** Antibodies used in this study.

Antibody	Species	Catalog number	Company
CRMP2	Rabbit	C2993	Sigma, St. Louis, MO
CRMP2 p32	Rabbit	generously provided by Dr. Yoshio Goshima (Yaksh and Rudy, 1976)	
CRMP2 p479	Rabbit	generously provided by Dr. Pascale Giraudon (Yang et al., 2014)	
CRMP2 p509/p514	Sheep	PB-043	Kinasource, Dundee, Scotland, UK
CRMP2 p522	Rabbit	CP2191	ECM Biosciences, Versailles, KY
CRMP2 p555	Rabbit	CP2251	ECM Biosciences, Versailles, KY
βIII-Tubulin	Mouse	G712A	Promega, Madison, WI
Synaptophysin	Mouse	MAB5258	Thermofisher Scientific
PSD95	Mouse	MA1-045	Thermofisher Scientific
Flotillin	Rabbit	F1180	Sigma, St. Louis, MO

### 2.4 Synapse enrichment and fractionation

Adult rats were killed by isofluorane overdose and decapitation, the spinal cords dissected, the lumbar region isolated and separated into contralateral and ipsilateral sides. Only the dorsal horn of the spinal cord was used as this structure contains the synapses arising from the DRG. Synaptosomes isolation was done according to (Pacchioni et al., 2009). Fresh tissues were homogenized in ice-cold Sucrose 0.32 M, HEPES 10 mM, pH 7.4 buffer. The homogenates were centrifuged at 1000×*g* for 10 min at 4 °C to pellet the insoluble material. The supernatant was harvested and centrifuged at 12,000×*g* for 20 min at 4 °C to pellet a crude membrane fraction. The pellet was then re-suspended in a hypotonic buffer (4 mM HEPES, 1 mM EDTA, pH 7.4) and the resulting synaptosomes pelleted by centrifugation at 12,000×*g* for 20 min at 4 °C. The synaptosomes were then incubated in 20 mM HEPES, 100 mM NaCl, 0.5% triton X, pH = 7.2) for 15 min on ice and centrifuged at 12,000×*g* for 20 min at 4 °C. The supernatant was considered as the non-postsynaptic density (non-PSD) membrane fraction, sometimes referred to as the triton soluble fraction. The pellet containing the postsynaptic density fraction (PSD) was then solubilized (20 mM HEPES, 0.15 mM NaCl, 1% triton X100, 1% deoxycholic acid, 1% SDS, pH = 7.5). The integrity of non-PSD and PSD fractions was verified by immunoblotting for PSD95, which was enriched in PSD fraction, and synaptophysin which was enriched in non-PSD fraction (see Fig. 2A). All buffers were supplemented with protease and phosphatase inhibitor cocktails. Protein concentrations were determined using the BCA protein assay.

### 2.5 Indwelling intrathecal catheter

Rats were anesthetized (ketamine/xylazine anesthesia, 80/12 mg/kg i.p.; Sigma-Aldrich) and placed in a stereotaxic head holder. The cisterna magna was exposed and incised, and an 8-cm catheter (PE-10; Stoelting) was implanted as previously reported, terminating in the lumbar region of the spinal cord (Yaksh and Rudy, 1976). Catheters were sutured (3-0 silk suture) into the deep muscle and externalized at the back of the neck; skin was closed with autoclips. After a recovery period of 5–7 days after implantation of the indwelling cannula, the spared nerve injury was induced.

### 2.6 In vivo transfection

For in vivo transfection, the plasmids pdsRed2-N1-empty vector, pdsRed2-N1-CRMP2, pdsRed2-N1-CRMP2-S522A or pdsRed2-N1-CRMP2-S522D were from (Dustrude et al., 2016). CRMP2 siRNA (5′-GTAAACTCCTTCCTCGTGT-3′) (Brittain et al., 2009) or siRNA Control (Cat# 12935300) were obtained from Thermo Fisher Scientific, Waltham, MA. Plasmids were diluted to 0.5 µg/µl in 5% sterile glucose solution and siRNA were diluted to 6 µM in 5% sterile glucose solution. Then, Turbofect in vivo transfection reagent (Cat# R0541, Thermo Fisher Scientific, Waltham, MA) was added at 1/17 dilution. For injections, 15 µL indicated plasmid complexes (n = 6/group) were injected into the intrathecal space followed by a 5 µL saline flush.

### 2.7 Mechanical allodynia

Rats were allowed to acclimate within suspended wire mesh cages for 30 min prior to behavioral assessment. Before (pre-baseline), after SNI (post-baseline) and 3, 24, 48, 72 h time points were used to measure response to calibrated von Frey filaments (g) probed perpendicular to the lateral plantar surface of the left hind paw (up-down method). Paw withdrawal thresholds were calculated in grams using the Dixon non-parametric test and expressed as the Paw Withdrawal Threshold (mean ± standard error; SEM) in GraphPad Prism 6.0. All behavior experiments were blinded.

### 2.8 Statistical analyses

Differences between means were compared by either paired or unpaired two-tailed Student’s t-tests. Behavioral threshold values were statistically analyzed for each foot separately and the significance of differences between the average of at least two pre-injection tests and the mean obtained for each post-injection test. In all tests, baseline data were obtained for the SNI and sham-treated groups before plasmid administration. Within each treatment group, post-administration means were compared with the baseline values by repeated measures analyses of variance (RMANOVA) followed by post hoc pairwise comparisons (Student-Newman-Keuls). A p value of <0.05 was used to indicate statistical significance between treatment and non-treatment groups.

## 3 Results

### 3.1 CRMP2 phosphorylation levels are dysregulated in neuropathic pain

**Fig. 1:**
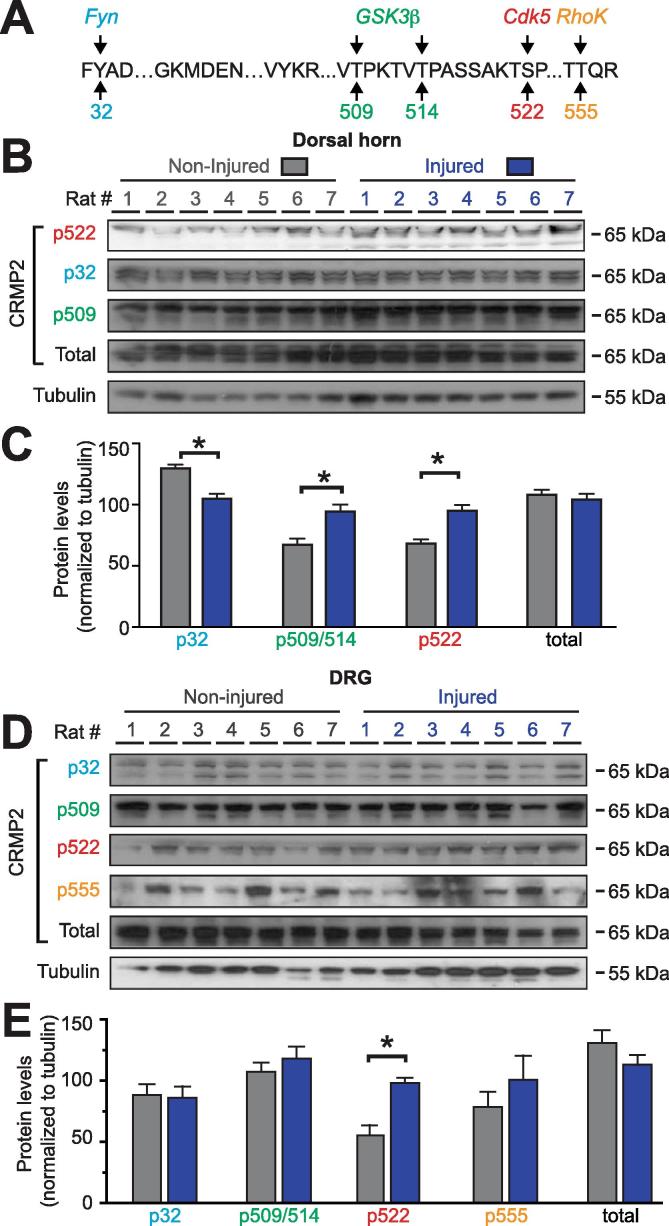
Alterations of CRMP2 phosphorylation levels in a rodent model of spared nerve injury (SNI). (A) Amino acid sequence of rat CRMP2 (Universal Protein Resource #P47942 (rat)) identifying the kinases (Src-family kinase Fyn (*blue*), glycogen synthase kinase 3β (GSK3β; *green*), cyclin-dependent kinase 5 (Cdk5; *red*), and Rho-associated protein kinase (ROCK; *orange*) and their target phosphorylated sites (numbers refer to the amino acid modified by the kinases). Representative immunoblots of spinal cord dorsal horn (B) or dorsal root ganglia (D) from spared nerve injury (SNI) rodents, ipsilateral (injured) and contralateral (non-injured) sides (n = 7), probed with phosphorylation-specific CRMP2 antibodies. No signals were detected for CRMP2 phosphorylated by the Src-family kinase Yes (Y479). Quantitative analyses of CRMP2 phosphorylation levels in ipsilateral (injured) and contralateral (non-injured) sides from SNI rodents (C, E, G, I). Tubulin was used as a loading control. Data show means ± s.e.m. of seven animals. *p < 0.05; Kruskal-Wallis test with Dunnett’s *post hoc* comparisons. (For interpretation of the references to colour in this figure legend, the reader is referred to the web version of this article.)

In nerve injury-induced neuropathic pain, the afferent input generated by the injury and intense noxious stimuli triggers an increased excitability of nociceptive neurons in the spinal cord. To determine where along this nociceptive pathway CRMP2 expression and phosphorylation levels are altered, we used a western blot approach on glabrous skin (contains nerve terminals), sciatic nerve, dorsal root ganglion (DRG; harbors the neuronal soma), and the dorsal horn of the spinal cord (contains the synapse from the primary afferents). CRMP2 can be phosphorylated by 5 kinases (Fyn, Yes, Cdk5, GSK3β and RhoK) at identified amino-acids residues (Fig. 1A). Using specific antibodies against CRMP2 and its phosphorylated forms (Table 1), we quantified CRMP2 expression and phosphorylation levels in tissues from the ipsilateral (painful) side of rats subjected to a spared nerve injury (SNI) compared to the contralateral (non-painful) side of the same animal. All animals were validated for expression of mechanical allodynia prior to western blot analyses. In all tissues tested, total CRMP2 expression remained unchanged between the injured and non-injured sides (Fig. 1) and no specific signal was detected for CRMP2 phosphorylation by Yes (p479, where p refers to the phosphorylated residue). In the dorsal horn of the spinal cord, CRMP2 phosphorylation by Cdk5 (p522) and GSK3β (p509/p514) was increased while CRMP2 phosphorylation by Fyn (p32) was decreased (Fig. 1B, C) in injury compared to control. No signal was detected for CRMP2 p555. In DRGs (L4, L5 and L6 were pooled to ensure sufficient protein quantities), we found no significant change of CRMP2 phosphorylation levels for p32, p555 and p509/p514 (Fig. 1D, E). CRMP2 phosphorylation by Cdk5 was increased in the injured side compared to the non-injured side (Fig. 1D, E). Collectively, these results identify CRMP2 expression and phosphorylation in tissues involved in pain signal transmission. Most notably, CRMP2 phosphorylation is dysregulated after SNI: CRMP2 phosphorylation at site S522 (Cdk5) was increased in dorsal horn of the spinal cord, and DRG.

### 3.2 CRMP2 phosphorylation (p522) is increased at the pre-synaptic sites of the dorsal horn of the spinal cord

**Fig. 2:**
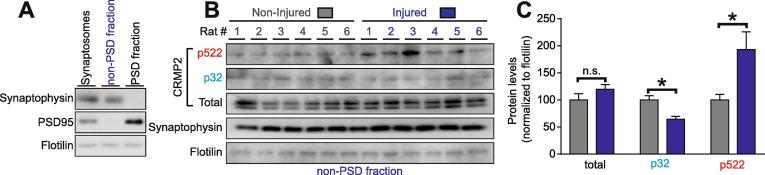
CRMP2 phosphorylation is enhanced at the presynaptic sites of the dorsal horn. (A) Immunoblots showing the integrity of the synaptic fractionation from lumbar dorsal horn of the spinal cord. The non-PSD fraction was enriched in the pre-synaptic marker Synaptophysin and the PSD fraction was enriched in the post-synaptic marker PSD95. The membrane-associated protein flotillin was used as a loading control. (B) Immunoblots showing the pre-synaptic CRMP2 expression, CRMP2 p32 and CRMP2 p522 levels in the lumbar dorsal horn of the spinal cord of animals having received a spared nerve injury. Synaptophysin shows the consistency of the pre-synaptic material analyzed. Flotilin is used as a loading control. (C) Bar graph showing decreased CRMP2 p32 and increased CRMP2 p522 levels at the pre-synaptic sites in the ipsilateral side of lumbar dorsal horn of the spinal cord. Mean ± s.e.m., *p < 0.05, Mann-Whitney compared to the contralateral side. PSD: post-synaptic density.

CRMP2 is expressed at synapses and contributes to dendritic spine density (Tobe et al., 2017) and synaptic bouton size (Brittain et al., 2009). To test if CRMP2 p522 is increased within pre-synaptic sites of the dorsal horn, we used a biochemical approach to purify synaptosomes from the dorsal horn of the spinal cord, followed by a fractionation step to isolate the post synaptic density (PSD) from the pre-synaptic (non-PSD) fraction (Fig. 2A). This allows us to assess CRMP2 phosphorylation levels in the synapses arising from the DRG to the spinal cord and to isolate contralateral (non-injured) versus ipsilateral (injured) sides from SNI rats (Fig. 2B). First, CRMP2 expression levels were analyzed in non-PSD fraction compared to the PSD fraction (Supplementary Fig. 1A). This revealed that CRMP2 expression and phosphorylation are mainly expressed within the pre-synaptic sites in the dorsal horn of the spinal cord (Supplementary Fig. 1B). Pre-synaptic fractions were probed for CRMP2 expression levels and, consistent with our previous data (Fig. 1C), no significant difference in total CRMP2 levels was detected between control and injured sides of SNI rats (Fig. 2C). Because CRMP2 p32 levels were decreased in whole dorsal horn lysates (Fig. 1C), we tested if this effect was localized to the pre-synaptic site. Indeed, CRMP2 phosphorylation levels by Fyn were decreased at pre-synaptic sites of the ipsilateral side of the dorsal horn (Fig. 2C). We next tested if CRMP2 p522 levels were enriched in these fractions. We found an increase of CRMP2 phosphorylation levels by Cdk5 at pre-synaptic site on the ipsilateral side compared to the contralateral side of SNI rats (Fig. 2C), in agreement with a similar increase noted in whole spinal cord lysates (Fig. 1C). These results show that CRMP2 phosphorylation changes in SNI are localized to pre-synaptic sites and could underlie neuronal plasticity following injury that may lead to neuropathic pain.

### 3.3 CRMP2 expression is necessary for neuropathic pain

**Fig. 3:**
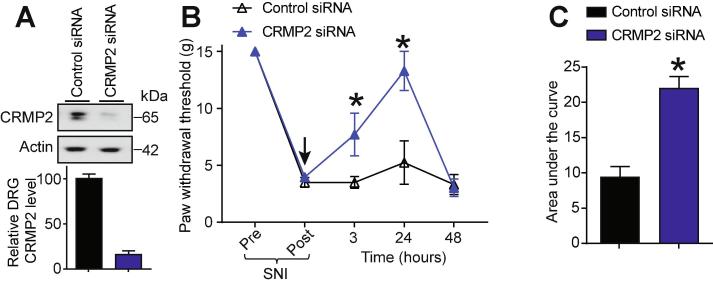
SNI mediated allodynia requires CRMP2 expression. (A) Representative immunoblot and bar graph showing decreased CRMP2 expression in DRGs, 24 h after CRMP2 siRNA intrathecal injection. Data is shown as mean ± SEM, n = 6, *p < 0.05, Mann-Whitney compared to control siRNA. (B) Rats developed allodynia 7 days after SNI. Intrathecal injection (*black arrow*) of CRMP2 or Control siRNAs significantly reversed the mechanical allodynia, measured by paw withdrawal threshold, at 3 and 24 h after injection (n = 6; *p < 0.05 vs. pre-injection baseline, 2-way ANOVA, post hoc Student-Neuman-Keuls). (C) Summary of data shown in panel (B) as area under the curve (AUC) for 0–48 h. Mean ± s.e.m., n = 6, *p < 0.05 versus control siRNA, Mann-Whitney.

Our results suggest that CRMP2 expression is an important component of neuropathic pain. To test if CRMP2 could control nociceptive behaviors after SNI, rats having received a SNI (day 7) were injected (intrathecal, i.th.) with CRMP2 or control siRNA (Brittain et al., 2009) complexed with TurboFect in vivo for in vivo siRNA transfection. This resulted in decreased CRMP2 protein expression at 24 h after intrathecal injection (Fig. 3A). Starting at 3 h and lasting until 24 h after injection of CRMP2 siRNA, nociceptive behaviors (paw withdrawal thresholds) were reversed in SNI rats (Fig. 3B). Allodynic behavior was restored at 48 h consistent with the turnover of CRMP2 (∼6 h half-life) over this period (Balastik et al., 2015, Moutal et al., 2017) and a limitation of the non-viral transfection method used here. Control siRNA-injected rats did not show any change in their paw withdrawal threshold (Fig. 3B). We calculated the area under the curve (AUC) to assess the effect of each treatment over the full experimental duration (Fig. 3C) and found a significant reversal of SNI-induced allodynia following CRMP2 knockdown in vivo. This data shows that CRMP2 expression is *necessary* for neuropathic pain.

### 3.4 Genetic targeting of CRMP2 phosphorylation by Cdk5 reverses neuropathic pain.

**Fig. 4:**
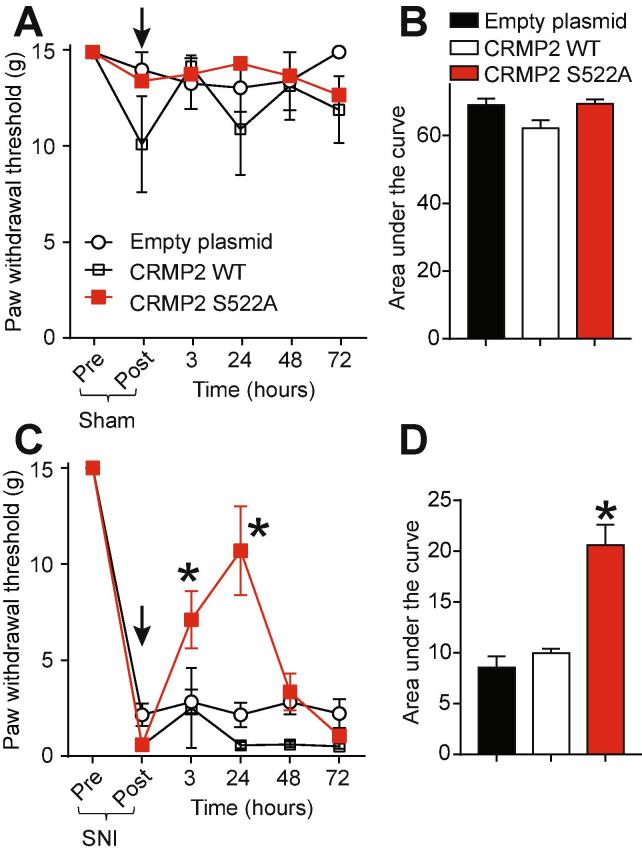
CRMP2 loss of phosphorylation reverses mechanical allodynia following SNI. (A) Paw withdrawal thresholds for sham-injured rats spinally administered (*black arrow*) with dsRed (empty plasmid), CRMP2 wildtype (WT) or CRMP2 S522A (15 µg/rat; intrathecal; n = 5–6). (B) Summary of data shown in panel (A) plotted as Area Under the Curve (AUC) for 0–72 h. (C) Paw withdrawal thresholds (PWTs) for rats having an SNI injury and spinally administered (*black arrow*) with empty, CRMP2 or CRMP2 S522A plasmids (15 µg/rat, intrathecal; n = 9–10). (D) Summary of data shown in panel (C) plotted as AUC for 0–72 h. *p < 0.05 compared to empty plasmid. SNI PWTs were measured on the lateral side of the paw.

CRMP2 phosphorylation by Cdk5 is increased in glabrous skin, DRG and dorsal horn following SNI (Fig. 1). Therefore, mimicking loss of CRMP2 phosphorylation by Cdk5 in vivo might be anti-nociceptive. To test this hypothesis, plasmids harboring a Cdk5-phospho-null CRMP2 (CRMP2 S552A) and controls (empty plasmid or CRMP2 wildtype (WT)) were evaluated for potential efficacy against SNI-induced mechanical allodynia in rats. Sham animals received an intrathecal injection of either empty (control), CRMP2 WT or CRMP2 S522A plasmids and then paw withdrawal thresholds (PWTs) were measured 3 h after injection and once a day for 3 days (Fig. 4A). Neither of the plasmid conditions altered the PWT of these animals as showed by the time-course of PWT (Fig. 4A) and the area under the curve (AUC) (Fig. 4B). This shows that in vivo plasmid transfection of CRMP2 WT or CRMP2 S522A has no effect on PWT in sham animals. SNI significantly reduced PWT 7 days post injury (Fig. 4C). At day 7 after the SNI, animals received a spinal administration of either empty (control), CRMP2 WT or CRMP2-S522A plasmids. While the injection of empty or CRMP2 WT plasmids had no effect on PWT, injecting CRMP2 S522A plasmid significantly increased PWTs over post-baseline SNI-values at 3 and 24 h post-injection compared to controls (Fig. 4C). We also determined the AUC to assess effects over the full experimental duration (Fig. 4D): CRMP2 S522A significantly reversed SNI-mechanical allodynia compared to empty and CRMP2 WT plasmids. These findings demonstrate that CRMP2 phosphorylation is *necessary* for neuropathic pain.

### 3.5 CRMP2 phosphorylation by Cdk5 is sufficient to elicit allodynia.

**Fig. 5:**
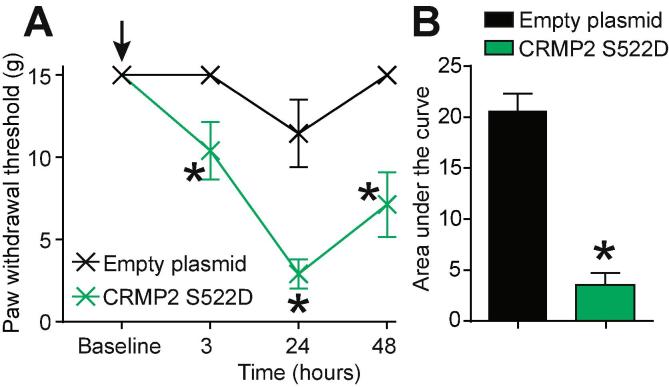
Expression of constitutively phosphorylated CRMP2 induces mechanical allodynia. (A) Paw withdrawal thresholds for naïve rats spinally administered (*black arrow*) with empty plasmid or CRMP2 S522D plasmids (15 µg/rat; intrathecal; n = 6). (B) Summary of data shown in panel (A) plotted as AUC for 0–48 h. Data is shown as mean ± s.e.m., and was analyzed by non-parametric two-way analysis of variance where time was the within subjects factor and treatment was the between subjects factor (ANOVA; post hoc: Student-Neuman–Keuls); AUCs were compared by Mann-Whitney non-parametric test.

Thus far, our results have established that CRMP2 protein expression and phosphorylation by Cdk5 are necessary for neuropathic pain. Our results also identified the upregulation of CRMP2 phosphorylation by Cdk5 in neuropathic pain. We demonstrated that expressing CRMP2-WT or CRMP2-S522A (resistant to phosphorylation) in non-injured rats does not produce nociceptive behaviors (Fig. 5A). We next asked if CRMP2 phosphorylation by Cdk5 is sufficient to elicit a nociceptive behavior. To test this, we injected (i.th.) naïve rats with either an empty plasmid or a plasmid expressing CRMP2 carrying a S522D mutation. This aspartate mutation mimics CRMP2 constitutive phosphorylation on serine 522 (Dustrude et al., 2016). At 3 h after injection, naïve rats injected with CRMP2-S522D developed allodynia while rats injected with an empty plasmid did not (Fig. 5A). This effect peaked at 24 h after injection and lasted until 48 h (Fig. 5A). The area under the curve was significantly reduced in rats injected with CRMP2-S522D plasmid compared to rats injected with the empty plasmid (Fig. 5B). These results show that mimicking CRMP2 constitutive phosphorylation in vivo is *sufficient* to elicit a nociceptive behavior.

## 4 Discussion

The results reported here identify CRMP2 phosphorylation by Cdk5 to be an intrinsic pathological event participating in the establishment of chronic neuropathic pain. We found this particular CRMP2 phosphorylation to be increased in DRG neurons and in the pre-synaptic sites of the dorsal horn of the spinal cord in neuropathic pain. Finally, we used genetic approaches to control CRMP2 expression or phosphorylation levels in vivo. We identified that CRMP2 expression and phosphorylation on serine 522 were required for mechanical allodynia in SNI. We also found that the gain of CRMP2 phosphorylation on serine 522 produces allodynia in naïve rats. Our findings highlight CRMP2 phosphorylation by Cdk5 as an important target for the control of neuropathic pain.

We first characterized CRMP2 phosphorylation pattern in tissues participating in the pain pathway. Here, only CRMP2 phosphorylation by Cdk5 was consistently upregulated. CRMP2 phosphorylation by Cdk5 occurs on serine 522 and is a known priming event for additional post-translational modifications. Indeed, CRMP2 phosphorylation by GSK3β requires prior phosphorylation by Cdk5 (Cole et al., 2006). Increased GSK3β phosphorylated CRMP2 was observed in dorsal horn where Cdk5 phosphorylated CRMP2 was also augmented. Another post-translational modification regulating CRMP2 function is SUMOylation (Dustrude et al., 2013). CRMP2 SUMOylation too relies on prior phosphorylation by Cdk5 (Dustrude et al., 2016) and is increased in neuropathic pain (Moutal et al., 2017). CRMP2 SUMOylation is negatively regulated by CRMP2 phosphorylation by Fyn (Dustrude et al., 2016). In the dorsal horn of the spinal cord, we observed a concomitant decrease of CRMP2 p32 (Fyn) and an increase of CRMP2 p522 (Cdk5), which was enriched at the pre-synaptic sites. These alterations of CRMP2 phosphorylation status both result in increased CRMP2 SUMOylation (Dustrude et al., 2016) and provide a molecular basis for the increased CRMP2 SUMOylation observed in neuropathic pain (Moutal et al., 2017).

Cdk5 is the key kinase responsible for phosphorylating CRMP2 (Khanna et al., 2012). Cdk5 has been previously associated with pain signaling (Pareek and Kulkarni, 2006). Cdk5 undergoes epigenetic upregulation in neuropathic pain (Li et al., 2014) and contributes to hyperalgesia (Yang et al., 2007). To become functional, Cdk5 needs to associate with p35 (Tang et al., 1995). Expression of p35 was directly related to nociceptive responses as mice deficient for p35 (p35^−/−^) developed an analgesic phenotype and mice overexpressing p35 (Tgp35) had a hyperalgesic phenotype (Pareek et al., 2006). Cdk5 activity can be increased (Hashiguchi et al., 2002) following p35 cleavage into p25 by calpain (Kusakawa et al., 2000). Increased p25 levels were reported in pain states (Pareek et al., 2006). Thus, Cdk5 activity in neuropathic pain is augmented by at least two distinct mechanisms: (*i*) increased gene transcription following epigenetic changes and (*ii*) increased activity because of p35 cleavage to its active co-factor p25. Another molecular event leading to increase in Cdk5 activity is the SUMOylation of p35/p25 (Buchner et al., 2015). Whether SUMOylation processes are dysregulated in neuropathic pain remains unknown but the increased CRMP2 SUMOylation found after a nerve injury (Moutal et al., 2017) suggests that it could be the case. Considering this, a positive feedback mechanism of upregulated SUMOylation increasing Cdk5 activity, resulting in increased CRMP2 phosphorylation and facilitation of subsequent CRMP2 SUMOylation could contribute to neuropathic pain.

We highlighted here a central role for the dysregulation of CRMP2 phosphorylation by Cdk5 in neuropathic pain. Post-translational modification(s) of CRMP2 induce a fundamental change of CRMP2′s cellular functions. For example, unphosphorylated CRMP2 binds to tubulin to promote neurite outgrowth (Fukata et al., 2002), whereas CRMP2 phosphorylation by Cdk5 decreases CRMP2′s affinity for tubulin and abolishes CRMP2 mediated neurite outgrowth (Cole et al., 2006). In turn, CRMP2 has enhanced binding affinity for CaV2.2 channels (Brittain et al., 2012) and positively regulates NaV1.7 channels (Dustrude et al., 2016). Both of these channels are involved in pain signal transmission by controlling neurotransmitter release and neuronal excitability (Tibbs et al., 2016). Consistent with these findings, we found that restricting CRMP2 phosphorylation by Cdk5 inhibits evoked action potential firing (Dustrude et al., 2016) and inhibition of the release of the nociceptive neurotransmitter CGRP (Moutal et al., 2016;1(1).). Another example of the contribution of CRMP2 phosphorylation to a nociceptive behavior was demonstrated in studies where CRISPR/Cas9-editing of *Nf1* gene (which codes for the protein neurofibromin) resulted in increased NaV1.7 and CaV2.2 currents; increased action potential firing (Moutal et al., 2017); increased CGRP release; and hyperalgesia (Moutal et al., 2017). The common denominator in these edited rats contributing to these dysregulations was increased CRMP2 phosphorylation by Cdk5 which was due to loss of CRMP2′s interaction with neurofibromin (Moutal et al., 2017). This interaction also regulates CRMP2′s interaction with syntaxin1A, a protein involved in docking of synaptic vesicles with the presynaptic membranes (Khanna et al., 2007), which is required for CGRP release and nociceptive signal transmission (Moutal et al., 2017). Our data showing increased CRMP2 phosphorylation in neuropathic pain links these studies together by demonstrating a key pathological event underlying increased ion channel trafficking, ectopic neuronal firing, augmented CGRP release and nociceptive behaviors. Consistently, we detected more phosphorylated CRMP2 at pre-synaptic sites within the dorsal horn of the spinal cord. We propose here a model where phosphorylated CRMP2 participates in the assembly of a pre-synaptic nociceptive signaling platform containing NaV1.7, CaV2.2, syntaxin1A and CGRP containing vesicles.

The SNI model of neuropathic pain relies on an axotomy and a ligation of the severed nerves (Decosterd and Woolf, 2000). After an axotomy the nerves undergo a degenerative process leading to multiple biological molecules to be released in the extracellular space. CRMP2 was found to be secreted by degenerating sciatic nerves (Castillo et al., 2018). The CRMP2 found in conditioned media from cultured sciatic nerves can trigger a calcium influx through CaV2.2 and NMDA receptor activation in hippocampal neurons but also in DRG neurons (Castillo et al., 2018). This suggests that additionally to its well-known function in ion channel trafficking, CRMP2 could act on the extracellular pool of ion channels and directly activate their function (Moutal and Khanna, 2018). While the phosphorylation status of this secreted CRMP2 remains unknown, the increased CRMP2 phosphorylation described here could be another element contributing to CRMP2 release into the extracellular space after an axotomy and raises the question of the function for extracellular phosphorylated (p522) CRMP2 contribution to neuropathic pain.

Targeting of CRMP2 phosphorylation to treat neuropathic pain appears as a potential therapeutic strategy is supported by findings which demonstrate that (*i*) CRMP2 expression is *required* for neuropathic pain; (*ii*) loss of CRMP2 phosphorylation (i.e., expression of a CRMP2 mutated on Cdk5 phosphorylation site (S522A)) is *sufficient* to reverse neuropathic pain; and (*iii*) gain of CRMP2 phosphorylation (i.e., expression of a phosphorylation-mimicking CRMP2 mutant (S522D)) is *sufficient* to reverse neuropathic pain. In order to achieve inhibition of CRMP2 phosphorylation, an obvious strategy is inhibition of the phosphorylating kinase Cdk5. Cdk5 inhibition has been previously tried for the treatment of neuropathic pain (Yang et al., 2014) but Cdk5′s numerous other functions (Shah and Rossie, 2017) render this strategy unlikely to reach acceptable pain relief in absence of unacceptable off-target effects such as memory impairment (Li et al., 2001, Sherry and Crowe, 2008). An alternative strategy is identification of molecules that specifically inhibit CRMP2′s phosphorylation by Cdk5. We identified the small molecule (*S*)-lacosamide as a specific inhibitor of CRMP2 phosphorylation by Cdk5 (Moutal et al., 2016). Inhibition of CRMP2 phosphorylation with (*S*)-lacosamide was beneficial in preclinical models of chronic pain including SNI (Moutal et al., 2016), headache (Moutal et al., 2016;1(1).), neurofibromatosis type 1 related pain (Moutal et al., 2017), and even glioblastoma (Moutal et al., 2017). Our novel findings that CRMP2 phosphorylation levels are increased in neuropathic pain suggests that the observed efficacy of (S)-lacosamide for chronic pain treatment is due to the specific targeting of a pathological event. Thus, we anticipate fewer off-target effects in non-disease states because of the low endogenous levels of CRMP2 phosphorylation. These studies underscore the therapeutic potential for targeting post-translational modifications of CRMP2 (phosphorylation by Cdk5, SUMOylation) for human disease, including neuropathies.

## 5 Summary

Neuropathic pain is dependent on CRMP2 expression and CRMP2 phosphorylation by Cdk5.
