# Supplementary material for: Cdk5-mediated CRMP2 phosphorylation is necessary and sufficient for peripheral neuropathic pain
Source: Neurobiol Pain. 2018 Jul 26;5:100022. doi: 10.1016/j.ynpai.2018.07.003 (PMC6505708; doi:10.1016/j.ynpai.2018.07.003)
Supplement: Supplementary data 1 [file mmc1.pdf]

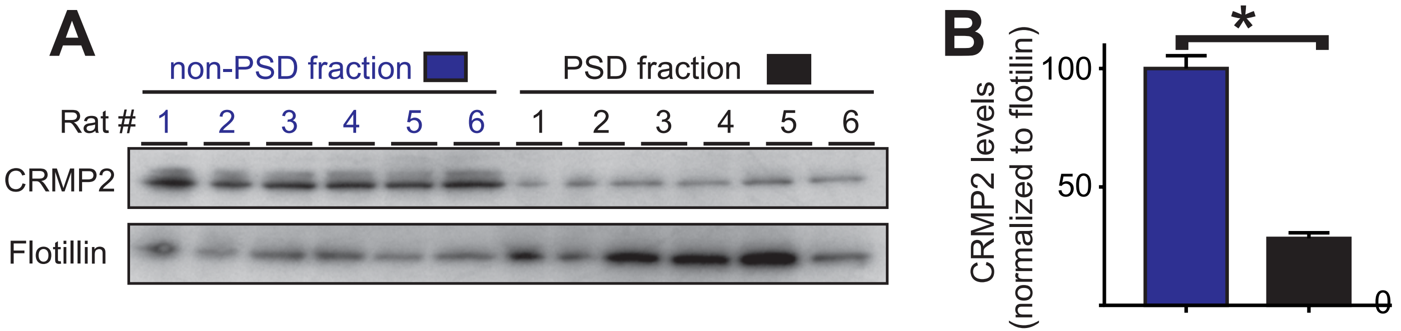

Supplementary Figure 1. CRMP2 expression is concentrated at the presynaptic sites of the dorsal horn. (**A**) Immunoblots showing the pre-synaptic and post-synaptic CRMP2 expression levels in the lumbar dorsal horn of the spinal cord of animals having received a spared nerve injury. Non-PSD fraction is pre-synaptic and PSD fraction is post-synaptic. Flotillin is used as a loading control. (**B**) Bar graph showing CRMP2 expression levels at the pre-synaptic sites compared to post-synaptic sites in the ipsilateral side of lumbar dorsal horn of the spinal cord. Mean ± s.e.m., *$p<0.05$, Mann-Whitney compared to the contralateral side. PSD: post-synaptic density.
